# A national cross-sectional study among drug-users in France: epidemiology of HCV and highlight on practical and statistical aspects of the design

**DOI:** 10.1186/1471-2334-9-113

**Published:** 2009-07-16

**Authors:** Marie Jauffret-Roustide, Yann Le Strat, Elisabeth Couturier, Damien Thierry, Marc Rondy, Martine Quaglia, Nicolas Razafandratsima, Julien Emmanuelli, Gaelle Guibert, Francis Barin, Jean-Claude Desenclos

**Affiliations:** 1 Infectious Diseases Departement, National Institute for Public Health Surveillance, Saint-Maurice, France; 2 Center for Research in Psychotropics, Health, Mental Health and Society, UMR CNRS 8136, University Paris Descartes, Inserm U611, Paris, France; 3 François-Rabelais University, Inserm ERI 19 and CHU Bretonneau, National Reference Center for HIV, Tours, France; 4 National Institute of Demographic Studies, Paris, France

## Abstract

**Background:**

Epidemiology of HCV infection among drug users (DUs) has been widely studied. Prevalence and sociobehavioural data among DUs are therefore available in most countries but no study has taken into account in the sampling weights one important aspect of the way of life of DUs, namely that they can use one or more specialized services during the study period. In 2004–2005, we conducted a national seroepidemiologic survey of DUs, based on a random sampling design using the Generalised Weight Share Method (GWSM) and on blood testing.

**Methods:**

A cross-sectional multicenter survey was done among DUs having injected or snorted drugs at least once in their life. We conducted a two stage random survey of DUs selected to represent the diversity of drug use. The fact that DUs can use more than one structure during the study period has an impact on their inclusion probabilities. To calculate a correct sampling weight, we used the GWSM. A sociobehavioral questionnaire was administered by interviewers. Selected DUs were asked to self-collect a fingerprick blood sample on blotting paper.

**Results:**

Of all DUs selected, 1462 (75%) accepted to participate. HCV seroprevalence was 59.8% [95% CI: 50.7–68.3]. Of DUs under 30 years, 28% were HCV seropositive. Of HCV-infected DUs, 27% were unaware of their status. In the month prior to interview, 13% of DUs shared a syringe, 38% other injection parapharnelia and 81% shared a crack pipe. In multivariate analysis, factors independently associated with HCV seropositivity were age over 30, HIV seropositivity, having ever injected drugs, opiate substitution treatment (OST), crack use, and precarious housing.

**Conclusion:**

This is the first time that blood testing combined to GWSM is applied to a DUs population, which improve the estimate of HCV prevalence. HCV seroprevalence is high, indeed by the youngest DUs. And a large proportion of DUs are not aware of their status. Our multivariate analysis identifies risk factors such as crack consumption and unstable housing.

## Background

Drug users (DUs) are at high risk for HIV and HCV infections [1]. Among DUs, HCV, like HIV, is mainly transmitted through needle sharing. However, HCV (Hepatitis C Virus) is more easily transmitted than HIV because its prevalence is greater than for HIV (Human Immunodefiency Virus), it is more resistant to desiccation [2,3] and is also linked to the sharing of drug paraphernalia (water, filter, spoon) [4-8]. HCV infection can, therefore, occur after much less injection and sharing episodes [9-12]. To minimize the risk of HIV and HCV, harm reduction policies have been adopted in many industrialized countries [13-17]. Many studies have studied the epidemiology and risk factors for HCV infection among DUs [18-27] and shown that harm reduction policies have had a major impact on HIV among DUs but a much more limited impact on HCV infection [10,28-36]. HCV prevalence estimates remain high [37], over 50% in the majority of countries where data are available [38]. In all these studies, prevalence and sociobehavioural data among DUs are available and useful for public health issues. So far, no study has taken into account in the sampling weights one important aspect of the way of life of DUs, namely that they can use one or more specialized services during the study period. This has an impact on their inclusion probabilities, and accordingly on estimates.

In France, most data on HIV and HCV infection among DUs come from notifications or highly selected population samples [39-42] and no prevalence estimates of HIV and HCV infection based on laboratory confirmation were available among DUs. In 2004–2005, we therefore conducted a national survey among DUs who resided in metropolitan France (the ANRS-Coquelicot study) to assess the prevalence of HIV and HCV infections based on serum testing, the frequencies of at risk practices, and the risk factors for HCV prevalence. To improve the estimate of HIV and HCV prevalences, we used a sampling design including the Generalised Weight Share Method (GWSM) described by Ardilly and Lavallee [43,44], taking into account the use of specialized services by each DU during the study period.

## Methods

In 2004–2005, we conducted a two stage random survey of DUs in five French large cities (Lille, Strasbourg, Paris, Bordeaux and Marseilles). Given the expected size of the sample, these five cities were chosen due to the important number of specialized structures for DUs, and the prevalence of drug use in each city. Prevalence rates of drug addiction were estimated from available data on opiate substitution treatments (OST) [31]. The five cities from five different regions were also selected to represent the diversity of drug addiction (products and modes of consumption) in France. The first stage of the design was constituted of high-and low-threshold care structures for DUs and of general practitioners (GPs) and the second stage by DUs. A DU to be included in the survey was a person >18 years who had injected or snorted drugs "at least once in its life" and accepted to participate in the survey after informed consent.

### Sampling

The sampling of individuals is similar to the time-location sampling [45]. In each city we did an exhaustive inventory of all structures that provide services to DUs: accommodation services including residential centers, hotel rooms, "sleep-in"; drug treatment centers including methadone maintenance or psychotherapy; low threshold services including needle exchange programs and outreach work teams. We then constructed a sampling frame by half-day that structures were open. Pairs (structure/half-day) were selected using an unequal probability sampling. Inclusion probabilities were proportional to the structure's active DUs list to obtain a calendar of half-day visits of structures for DUs selection, inclusion and data collection. In each structure/half day visit, DUs present were selected using a simple random sampling, except for residential centers where all users were included. For GPs we first constituted a random sample of GPs stratified by the volume of substitution treatment prescribed (high versus moderate). Then, inclusion was proposed to all the DUs followed by the selected GPs.

### Subject inclusion and data collection

During each structure/half day visit, a number of DUs corresponding to the calculated sample size were asked to participate and included if they consented to the interview and self fingerprick blood sampling on dried blood spots for HIV and HCV testing. The questionnaire was administered over 30 to 40 minutes by professional interviewers independent of the recruitment structures.

Interviewers had been trained for DUs interview in order to minimize the social desirability bias with respect to drug consumption and at risk practices. The questionnaire explored DU'socio-demographic situation, health status, access to HCV screening, knowledge of HCV transmission modes, drug use, and at risk practices. Socio-demographic variables were related to the situation at the time of the study, such as for the level of education, employment, houselhold and living conditions, marital status. Other items investigated different period of DUs' lives such as ever previous jail experience, living with their families or not during adolescence. Health related items included knowledge of current HIV and HCV serological status, medical treatments, circumstances of last HCV or HIV testing and access to HCV treatments. Perception of health status and main health problems were investigated for the 6 months prior to interview. Drug consumed, modes of consumption (injecting, sniffing, smoking) and at-risk practices (sharing syringes, other injection materials, sniffing material or glass pipes) were recorded for the last month using the Injecting Risk Questionnaire (IRQ) [46]. DUs were also questioned on their injection and sniffing history over their whole lives. We also collected information on knowledge of HIV, HCV and HBV transmission modes.

### Laboratory methods

To obtain seroprevalence data, we analyzed blood samples on blotting papers. The dried blood spots (DBS) were cut out with a punch to obtain a circle of about 6 mm diameter, which was placed in 250 μl of 0.01 M phosphate sodium buffer containing 10% bovine serum albumin and 0.05% Tween 20 (PBS-BSA-TW) then incubated at room temperature for one hour in an ultrasonic cleaner. The eluted serum samples were directly used to fill the wells of ELISA microplates (200 μL per well). The above steps were performed strictly as recommended by the manufacturer. The third-generation ELISA kit from Ortho-Clinical Diagnostics (HCV 3.0, Raritan, NJ) was used to detect anti-HCV antibodies. The same procedure was used to detect HIV antibodies using the Ortho HIV1/2 Ab capture Elisa kit. Any anti-HIV positive sample was confirmed by serotyping and/or western blot [47].

We assessed the validity of the ELISA technique applied to DBS by comparing absorbance values for various serum samples positive for anti-HCV antibodies or anti-HIV antibodies, either tested directly as serum and as DBS eluates. The data showed an excellent concordance of results except for weakly positive sera from patients with resolved HCV for whom HCV-antibodies could be missed. Thus prevalence rates of HCV infection based on the DBS method must be considered as minimal estimates (see appendix - Additional file 1).

### Ethical considerations

Blood samples were collected, after informed consent, by means of fingerprick self-sampling. The individuals were informed that they would not receive the results of the tests made from fingerprick blood sampled on DBS. However, they were informed on the benefits and places of HCV and HIV screening and treatment through leaflets and posters in the survey participating structures. In addition during interviews, interviewers stressed the benefit of screening and directed DUs to near by screening services. The study protocol was approved by an ethical review board (Autorisation Number CCPPRB 02-002) because of its public health added value and the indirect individual benefit for participating DUs.

### Data analysis

For this kind of cross-sectional design, we classically assign a sampling weight to each individual to estimate the epidemiological indicators of interest (prevalence, odds ratio etc.) in the target population. The sampling weight is the inverse of the inclusion probability of a DU in the survey sample. The fact that DUs can use more than one structure during the study period has an impact on these inclusion probabilities. To calculate a correct sampling weight, we have to take into account the number of visits in participating structures during the course of the survey for each DU. We are in the general framework of an indirect sampling [44] in which there are two populations: the DU, noted U^A ^and the structures, noted U^B^, that are related to one another. We want to produce an estimate for U^B ^using the existing links between the two populations. To estimate an epidemiological indicator in a target population U^B ^using a sample selected from another population U^A ^is a major challenge if the links between the units of the two populations are not one-to-one because it becomes difficult to calculate the inclusion probabilities and consequently the sampling weights. A solution proposed by Ardilly and Lavallee is the generalised weight share method (GWSM) [43,44].

This technique is meant to be a methodological framework to estimate weights, including several weighting techniques used for different designs (longitudinal studies, network sampling, time-location sampling, snowball sampling, etc...). Time-location sampling is more widely described and used in the literature, particularly for sampling hard-to-reach or hidden population [45]. However, we introduce the GWSM as we assert that it is more appropriate than the time-location sampling because (1) the GWSM is more rigorous from a sampling theoretical point of view, while the TLS focuses on the probability of individuals' behaviour (model-based approach) and (2) the GWSM can account for several cases where the relationship between the sampling frame(s) and the target population is more complex.

To our knowledge, this method has not been yet introduced in epidemiology as a rigorous statistical method allowing to (1) calculate sampling weights and (2) encompass several methods more widely used and described in the literature.

**Figure 1:**
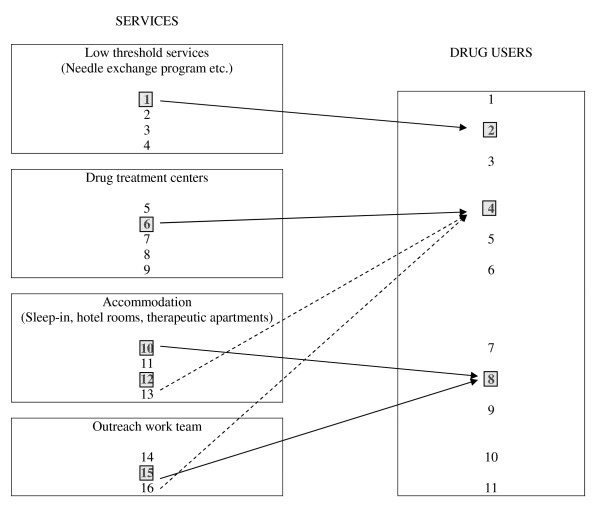
**Observed links (arrows) and declared links (dotted arrows) between the population of services and the population of drug users, ANRS-Coquelicot study, France 2004–2005**. The shaded boxes represent the sampled services and the sampled individuals.

This method produces a new weight for each unit in the target population U^B ^which is an average of the sampling weights of the population U^A ^from which the sample is selected. A fictitious sampling example is illustrated in Figure 1. The population U^A ^contains 16 structures and the population U^B ^contains 11 DUs. The shaded structures were sampled pointing to sampled DUs. The arrows indicate the links between the services and the DUs. Individual 1 reported a unique visit during the survey period. Individual 4 reported three visits in a sampled drug treatment center and in two non sampled services (accommodation n°13 and outreach work team n°16) represented by the dotted arrows. Individual 8 represents a fictitious situation in our study as it was almost impossible that a DU was interviewed several times. In practice, three questions were asked to DUs on the number of visits and the identification of structures visited to identify the links between the two populations. The GWSM needs inclusion probabilities  only for the selected units j in the sample s^A^. This is a major advantage in comparison with other methods based on exact calculation of selection probabilities of surveyed units. The classical sampling weights *w*_*i *_= 1/π_i _are first calculated for each individual i. Then the number of services visited by the individual i during the survey, noted r_i _and the number of times when the individual i has visited the service k during the survey, noted r_ik _were calculated. Finally a new weight is calculated by . For the illustrative example in figure 1, the new weights are , , .

All results reported in this article are estimates that take into account the two stage survey design including the GWSM. Statistical analyses were done with Stata 9 software. Comparisons of proportion were made with the chi-square test, with a significance threshold of 0.05. Prevalence ratio (PR) and their 95% confidence intervals (CI) were calculated to analyse univariate association between HCV status and risk factors. A multivariate logistic regression model was then used to identify independent risk factors for HCV seropositivity using a manual stepwise strategy. We also tested interactions between explanatory variables and no interaction was significant. Prevalence odds ratios are presented in our multivariate analysis, as it is more difficult to estimate prevalence ratios in a multivariate analysis taking into account the sampling design.

## Results

Among the 2389 DUs who were invited to participate in the survey, 483 refused, 444 did not correspond to the population of interest and 1462 accepted. The participation rate was 75%. There was no significant difference between those who participated and those who did not for age, gender and by type of services. Reasons of refusals included lack of time to respond (65%), psychological or physical status judged not compatible with the completion of a questionnaire (13%), lack of interest for the study (8.5%), refusal without reason (7.8%), 4.6% of refusals were not documented and only 3 refusals were linked to a care structures' staff.

Among DUs who participated, 79% accepted to provide a fingerprick blood sample. Reasons for not providing a blood sample were obtained on a sub sample of 53 refusals and included: lack of a confined space for collecting blood or no microlancet available (24.5%), thickness of finger skin (18.9%), refusal to self use the microlancet (20.8%), no time (13.2%), psychological or physical status judged not compatible with the test (11.3%) and religious reasons (3.8%). Only 3.8% persons refused by fears to be identified and 3.8% of refusals were linked to the non restitution of the test result. In multivariate analysis, the main factors associated to refusal of blood sampling were no prior HIV and HCV testing, knowing HCV transmission routes and having been recruited outside a general medical practice.

### Sociodemographic characteristics

Three-quarters of DUs were males. Mean age was 35.6 years for males and 34.5 years for females. Two-thirds of participants were unemployed. Only 45% of respondents had a stable household. The remaining 55% did not have their own home or did not live with a partner or their parents. Among these, 19% lived in a squat or in the street. About two thirds (61%) reported having been incarcerated at least once.

### HIV and HCV seroprevalence

**Figure 2:**
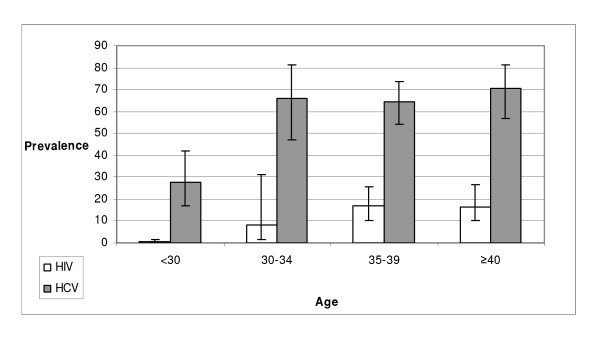
**HIV and HCV seroprevalence by age among French DUs, ANRS-Coquelicot study, France 2004–2005 (N = 817, DUs who consented to be tested)**.

The overall HIV and HCV seroprevalence were 10.8% [95% CI: 6.8–16.6] and 59.8% [95% CI: 50.7–68.3], respectively. Almost all HIV-seropositive DUs were also infected by HCV (HIV/HCV dual infection rate = 10.2% [95% CI: 6.3–15.9]). The HIV seroprevalence did not differ whether DU had ever injected drugs or not but increased gradually with age with only 0.3% of DU infected before the age of 30 years (figure 2) and differs among the five cities from 1% in Lille to 31,5% in Marseilles. There was no significant difference for HIV and HCV seroprevalence between males and females.

HCV seroprevalence did not differ significantly among the five cities included. Participants who had injected a drug at least once were more than 2.5 times more often seropositive for HCV (73.8%) than those who did not (27.9%). The proportion of HCV infection was 28% among those under 30 years of age and increased strongly after and was maximum over 40 years (71%, Figure 2). Analysis by five-year birth cohort (Table 1) indicates that HIV seroprevalence decreases in cohorts born after 1970 (less than 34 years old at the time of the survey), corresponding to DUs who could benefit from harm reduction services fully made available in France in 1994 (Syringe Exchange Programmes [SEP] and substitutive replacement therapy). For HCV, seroprevalence remained high in two 5-year birth cohorts born after 1970 and decreased for those born in 1980 and after (aged at least 24 years at the time of the survey).

**Table 1:** Seroprevalence for HIV and HCV among French DUs by 5-year birth cohort

Year of birth	% HIV+ [95% CI]	% HCV+ [95%CI]
<1959	17.3 [8.6–31.9]	65.9 [43.9–82.7]
1960–1964	16.4 [8.5–29.1]	72.1 [56.1–83.9]
1965–1969	16.7 [10.4–25.8]	64.5 [53.9–73.7]
1970–1974	8.2 [1.7–31.1]	66.2 [46.8–81.3]
1975–1979	0.0	35.1 [20.3–53.5]
1980–1984	1.1 [0.2–5.9]	11.1 [4.4–25.1]
1985–1989	0.0	0.0

* ANRS-Coquelicot study 2004–2005 (N = 817 DUs who consented to be tested)

### HCV screening, awareness of HCV status and HBV vaccination

More than 95% of respondents said they had already been tested at least once for HIV and 91% for HCV. While the lifetime HCV screening rate was high, the last negative test had been done 18 months previously on average. Moreover, 27% of DUs stated they were HCV-seronegative but they were HCV-seropositive according to biological tests. By comparison, only 2% of DUs wrongly said they were seronegative for HIV.

Vaccination coverage against HBV was 43% among DUs.

### Products consumed and at risk practices

In the month prior to interview, illicit psychoactive products consumed (whathever the mode of use) were crack/free base (30%), cocaine (27%), heroin (20%), and ecstasy (12%). Benzodiazepines had been consumed by 40% of respondents, hypnotics by 20% and amphetamines by 7%. In the previous six months, most respondents (71%) had received OST (Subutex^® ^in 57% of cases, methadone in 36%).

Ninety eight per cent of DUs said they had ever snorted drugs and 70% to have ever injected drugs. Mean age at first injection was 20.4 years with first injection done by another DU for 83%. In the last month, 40% of DUs reported they had injected at least once and among them 13% had shared a syringe (corresponding to 4% of all DUs), 38% other injection materials (filter, spoon, water, 11% of DUs) and 74% had reused their own syringe (21% of DUs). Crack users reported sharing often crack pipes (81% during the last month).

### Factors associated with HCV seropositivity

The complete list of significant variables in univariate analysis is presented in table 2. The following variables are only significant in the univariate analysis: HCV seropositivity was associated with having ever been jailed (prevalence ratio [PR] = 1.38), having a sexual partner drug user (PR = 1.30), subjective poor health (PR = 1.40), and fear of treatment for hepatitis C (PR = 1.33). Methadone use (PR = 1.34), morphine sulfates use (PR = 1.38) and having shared injection materials (PR = 1.54) in the last month were also risk factors for HCV in univariate analysis, while heroin use in the last month (PR = 0.59) and living in couple (PR = 0.73) were negatively associated with HCV. In multivariate analysis (table 3), factors that remained independently associated with HCV seropositivity were age over 30 (prevalence odds ratio [OR] = 3.4), HIV seropositivity (OR = 29.2), having ever injected drugs (OR = 9.2), OST in the last 6 months (OR = 3.2), crack smoking in the last month (OR = 2.7), and precarious housing (OR = 1.8).

**Table 2:** HCV prevalence by risk factors among French DUs*

		Prevalence of HCV	Prevalence Ratio	95% Confidence Interval	Missing data
Age	<30 years				
	≥ 30 years	67.45	2.43	2.04–3.60	4
Situation	Single	64.96			
	In couple	47.38	0.73	0.72–0.74	3
Type of household	Stable	50.90			
	Precarious	67.42	1.32	1.32–1.33	16
Ever been jailed	No	48.00			
	Yes	66.11	1.38	1.37–1.39	0
Percieved health (last 6 months)	Good	52.02			
	Bad	72.98	1.40	1.39–1.41	0
HIV serology	Negative	55.69			
	Positive	94.12	1.69	1.68–1.70	0
Fear of HCV Treatment	No	50.26			
	Yes	66.86	1.33	1.32–1.34	2
Injected at least once	No	27.90			
	Yes	73.82	2.65	2.58–2.71	0
Shared injection materials in the last month	No	55.96			
	Yes	86.17	1.54	1.53–1.55	2
Sexual partner drug user injector (last 6 months)	No	56.38			
	Yes	73.30	1.30	1.29–1.31	0
Heroine (last month)	No	64.76			
	Yes	38.11	0.59	0.57–0.60	0
Methadone (last month)	No	54.17			
	Yes	72.82	1.34	1.34–1.35	0
Moscontin (last month)	No	57.21			
	Yes	78.69	1.38	1.36–1.39	0
Crack (last month)	No	53.75			
	Yes	71.51	1.33	1.32–1.34	0
Substitution therapy (last 6 months)	No	31.77			
	Yes	67.75	2.13	2.07–2.20	0

* ANRS-Coquelicot study, France, 2004–2005 (N = 817 DUs who consented to be tested)

**Table 3:** Independent risk factors for HCV seropositivity*

Variable		Adjusted OR**	95% Confidence Interval
Age			
	<30 ans	1.00	
	≥ 30 ans	3.44	1.46–8.12
Type of housing			
	Stable	1.00	
	Precarious	1.80	0.99–3.27
HIV serology			
	Negative	1.00	
	Positive	29.17	9.79–86.87
Injected at least once			
	No	1.00	
	Yes	9.25	4.02–21.27
Crack smoking (last month)			
	No	1.00	
	Yes	2.65	1.19–5.91
Substitution therapy			
	No	1.00	
	Yes	3.24	1.01–10.37

* Multiple logistic regression analysis among French DUs. ANRS-Coquelicot study, France, 2004–2005 (N = 794)

**The reported prevalence odds ratios were adjusted for all the characteristics included in the table.

The multivariable analysis stratified by gender indicated some differences by gender. HCV seropositivity was associated with being HIV-positive (OR = 22.3), having ever injected drugs (OR = 10.4), being unemployed (OR = 3.5), history of abscesses in the last 6 months (OR = 4.8), having snorted drug in the last month (OR = 7.2), ever use of hypnotic drugs (OR = 0.1) or use of cannabis in the last month (OR = 0.3) among females. For males significant factors were having ever injected drugs (OR = 15,7), age over 30 years (OR = 9.6), no regular income (OR = 3), knowledge of HIV transmission modes (OR = 6.7), report of oedema of hands and feet in the last 6 months (OR = 3.4), use of methadone in the last month (OR = 2.3) and having smoked drugs in the last month (OR = 2.5), going to drug treatment centre the day before the study (OR = 0.4), prior HIV test (OR = 0.4) and consumption of heroin in the last month (OR = 0.4).

## Discussion

To our knowledge this is the first national survey among DUs, enabling estimates of HIV and HCV prevalence rates and their confidence intervals, based on a random sampling design using the Generalised Weight Share Method (GWSM) and blood testing. It is also the first attempt to apply the GWSM to a public health study. This method was previously used in a demographic survey of French homeless people [43] whose relationships with support facilities are almost similar to those of DUs. Our random sampling design allowed us to make inference to the French DUs reached by specialized services and GPs, and to obtain correct confidence intervals.

The use of GWSM with blood testing is a way to obtain more valid estimates of HCV prevalence. We showed that 27% of DUs were not aware of their HCV-seropositive status, probably because they had become infected since their last test. Therefore, HCV prevalence estimates based on declarative data do not reflect the real prevalence among DUs.

Because our study targeted DUs managed in specialized centers and by GPs, it does not provide information on "hidden" populations of DUs that do not use such services [48,49]. Furthermore, without available precise census data on the French DUs population, no post stratification could be done. In our study, the youngest, the most socially inserted and the females DUs are less likely to use DUs specialized structures which may have biased our estimates. However, better socially inserted DUs were partly included in the study through recruitment by GPs. A declaration bias by structures of the size of the population of DUs they care for is possible to increase public funding and could impact the sampling design. However, nothing allows us to assert that certain structures had overestimated DUs' visits more than others. We made an important effort to limit such biases by securing a high confidentiality of data collection and recording. A desirability bias is also possible as in all studies of stigmatized behaviours. To limit its impact we used professional interviewers who were totally independent of the investigated structures. Interviewers were trained and followed up during field data collection through meetings to maintain homogeneity, create a reliable climate towards DUs and to limit the social desirability bias. Although cities were not chosen at random, structures were included in 5 cities all over France to reflect the diversity of drug use in France and visit days in the structures and DUs were randomly selected. There were no differences by sex, age and service between participants and those who refused participation, however, no other data was available for comparison and guarantee comparability of those who refused and participated.

Despite these limitations, we believe that using this innovative statistical approach has improved the efficiency of our study and gave more valid estimates of the HIV and HCV public health burden among DUs and associated risk factors.

In the ANRS-Coquelicot study, the HCV seroprevalence (59.8%) is more than five times greater than for HIV (10.8%) and almost all those infected by HIV were also infected with HCV which stresses the huge public health burden of both infections in this population. HCV seroprevalence did not differ significantly among the five cities included, although HIV prevalence differed significantly between Lille (1%) and Marseilles (31.5%). This finding is probably related to differences in at-risk behaviour by city: in Marseille DUs are known to inject more (83% had ever injected) than DUs from other cities (65%). In the years 1970s to 1990s, DUs from south of France (including Marseille) injected heroin much more than in the North of France (including Lille) where heroin was more often smoked which is at much lower risk of HIV transmission.

Our analysis by year of birth indicates that the French harm reduction policy, fully implemented since 1994 (SEP and OST), had probably a strong impact on HIV transmission but much less and delayed for HCV transmission. We recognize that one must be careful when establishing a link between harm reduction programmes and their differentiated impact on HIV and HCV using a cross-sectional study. However, in France and in other western countries, the decrease of HIV prevalence among DUs, although partially explained by mortality, and the small proportion of DUs among HIV patients who recently discovered their HIV status observed since the implementation of harm reduction policies strongly suggest a link with harm reduction programmes [10,28-36].

The high HCV seroprevalence among DUs with about 30% of those under 30 years of age affected is a finding of high public health concern. It results from a combination of several factors, including the high prevalence of HCV infection (59.8%) in the global population, the higher transmissibility of HCV compared to HIV, and difficulties for DUs to reduce further behaviors at risk of HCV infection [50]. In addition to syringe and needles sharing, HCV is also transmitted by sharing drug paraphernalia (water, spoon, filter...) [4,7,8]. In our study the level of drug paraphernalia sharing (38%) remains substantial, and syringe reuse (74%) is very high. Reuse of a personal syringe can also be risky for HCV transmission if the drug solution is drawn from a shared container and can be considered as an "indirect sharing". The high HCV seroprevalence before 30 years old indicates that DUs are rapidly infected after drug initiation. New and young DUs are often initiated by older peers (83% in our study) who, because they are older, are more likely to be infected with HCV [51,52]. Furthermore, a third of DUs (27%) are not aware of their HCV seropositive status. HCV infection often occurs early after adoption of the intravenous route [9,12,53-55]. Therefore, the opportunity of HCV transmission will remain substantial without implementing new prevention strategies that target all these critical points.

Our multivariate analysis confirmed known risk factors for HCV infection, such as age, injection at least once and HIV seropositivity [23,56,57]. We also identified crack consumption and unstable housing conditions as risk factors. Social vulnerability and precarious housing conditions expose DUs to a greater risk of HCV transmission because they are dependent on others for injection, less able to anticipate the time of injection and prepare safely the necessary equipment. They may also have to inject in public places or in the homes of other DUs which favor unsafe practices and the sharing of injection equipment. However, precarious housing conditions may also be a consequence of HCV infection [58-60].

The link between HCV infection and crack smoking (in the last month) can be interpreted in two ways. In France, most crack users had injected heroine in the 1980s when no harm reduction policy was in place. Alternatively, sharing of crack pipes has been shown to be a possible way to transmit HCV [4,61,62]. Glass crack pipes become very warm and break which result in cuts and lip lesions that can serve as routes for HCV transmission [63-65]. Therefore, this association is interesting but complex to interpret. Indeed, it is difficult to say whether the association is due to crack users injection patterns in the past, whether it is due to transmission by crack pipes, or whether former injection drug users, many of whom could be infected by HCV, have switched to non-injection use of crack to try and lower their risk of infection with other pathogens.

Although the prevalence of HCV infection was similar by gender (56% among females vs 61% in males) it does not imply similar risk factors as shown in our analysis stratified by gender. While our results by gender must be interpretated cautiously because of model unstability (wide confidence intervals of OR), in a sociological analysis done within our study, women have been shown to be highly engaged in at-risk behaviours while consuming drugs or having sex [49]. Other studies have also reported differences in risk factors by gender among DUs, with females being more vulnerable than males [3,66-69].

In our study, questions on drug consumption and at-risk practices explored the month prior to interview. Therefore, the impact of long-term consumption and behaviours could not be assessed. It would have been useful to collect drug consumption over entire life to assess drug consumption trajectory of DUs. We chose not to include these variables in our questionnaire because the interview could not last more than 30 minutes in field conditions.

## Conclusion

The incidence of HCV infection in the most recent cohort study of DUs varied from 2% in the Netherlands in 2005 [70] to 10% in France in 2000 [71], 18% in the United States in 2000–2001 [72] and 42% in London in 2001 [33]. If most of the studies show that harm reduction policies have not yet had a perceptible impact on HCV transmission among DUs [73], a recent study in the Netherlands indicates that HCV transmission among DUs can be reduced by intense and large scale harm reduction policies combining access to SEP and opiate substitutive treatments [70,74] and that it takes longer to observe a measurable impact on HCV than on HIV. In France, it is too early to document a similar impact since the harm reduction policy was implemented later than in the Netherlands.

Our study shows that HCV seroprevalence is very high (59.8%) among DUs in France. The high frequency of third-party initiation in drug injection, the persistence of at risk behaviours, the lack of awareness of HCV serostatus, and the extreme social vulnerability facilitate HCV transmission, particularly for the youngest and most recent DUs. It is therefore necessary to focus prevention strategies on avoiding starting drug injection, sharing of injection paraphernalia and own reuse, and initiation of injection for both the initiator and the initiated. Additional preventive strategies are needed for crack users and educational programmes for safer injection of injectors. It is also important to take into account the health of DUs more globally through integrated social services and to improve access to screening and treatment of HCV.

## Abbreviations

ANRS: agence nationale de recherches sur le sida et les hépatites; CI: confidence interval; DBS: dried blood spot; DU(s): drug users(s); GPs: General Practioners; GWSM: Generalised Weight Share Method; HCV: hepatitis C virus; HIV: human immunodeficiency virus; OR: Odd Ratio; PR: prevalence ratio; OST: Opiate Substitution Treatment; SEP: syringe exchange programmes

## Competing interests

The authors declare that they have no competing interests.

## Authors' contributions

All authors read and approved the final manuscript. MJR is the main investigator for this research. She designed the survey, coordinated its implementation, data collection and scientific activites, conducted the main analysis and wrote the epidemiological section of the article. YLS prepared the sampling strategy of the survey, produced weighted parameter estimates and wrote the statistical section. EC merged the biological and epidemiological data and contributed to cleaning the database and to the preliminary analysis. DT did the biological analyses for the survey. MR and GG contributed to data analysis. MQ coordinated the field survey. NR contributed to the sampling strategy of the survey. JE designed the survey with MJR. FB designed the biological part of the survey and wrote the section on biological testing. JCD supervised the whole survey design and scientific work and edited the manuscript

## Pre-publication history

The pre-publication history for this paper can be accessed here:

http://www.biomedcentral.com/1471-2334/9/113/prepub

## Supplementary Material

Additional file 1 **Appendix**. Evaluation of the performance of the HCV EIA on dried blot spots. Click here for file
