# Supplementary material for: A national cross-sectional study among drug-users in France: epidemiology of HCV and highlight on practical and statistical aspects of the design
Source: BMC Infect Dis. 2009 Jul 16;9:113. doi: 10.1186/1471-2334-9-113 (PMC2733898; doi:10.1186/1471-2334-9-113)
Supplement: Additional file 1 — Appendix. Evaluation of the performance of the HCV EIA on dried blot spots. [file 1471-2334-9-113-S1.doc]

**Appendix**

**Evaluation of the performance of the HCV EIA on dried blot spots**

In order to assess the reliability of the ELISA technique applied to DBS we first spotted blood from three chronic HCV carriers on filter paper. The papers were allowed to dry overnight at room temperature and the DBS were then eluted as described above and serially diluted up to 1:100 000. The different dilutions were tested by ELISA in parallel with serial dilutions of fresh plasma collected at the same time. The eluates and their dilutions were directly used to fill the wells, whereas the plasma samples and their dilutions were used diluted, as recommended in the test procedure (20 µl of specimen in 200 µl of specimen diluent). As expected, analytical sensitivity was lower with samples spotted on filter paper. Indeed, two samples with absorbance values above the cut-off value at 10-3 dilution with fresh plasma were only positive at 10-1 dilution with the corresponding eluates, whereas a third sample with absorbance values above the cut-off value at 10-4 dilution with fresh plasma were only positive at 10-2 dilution with the eluate. However, strong signals were regularly obtained with the undiluted eluates (and even at 1:10 dilution), showing that the method was relevant for seroepidemiological studies. In a second set of experiments we analyzed the ability of the anti-HCV ELISA to identify various categories of anti-HCV-positive patients tested with the DBS method. We used serum samples from seven HCV RNA-positive patients strongly reactive with at least three HCV antigens in a recombinant immunoblot method (Chiron RIBA HCV 3.0 SIA (Ortho-Clinical Diagnostics, Raritan, NJ)), five HCV RNA-positive patients strongly reactive with only 1 or 2 HCV antigens by RIBA, seven HCV RNA-negative patients strongly reactive by ELISA and reactive to most HCV antigens by RIBA, and eight HCV RNA-negative patients weakly reactive by ELISA and even more weakly reactive by RIBA. The first two categories were representative of samples from chronically infected patients, whereas the latter two categories were representative of samples from patients with resolved infection. The dried serum spots were prepared with 20 µl of each sample, and the subsequent steps were performed as described above. All the eluted samples from the 12 HCV RNA-positive patients were clearly positive by ELISA (figure). Similarly, the eluted samples from the seven HCV RNA-negative patients that were strongly reactive by ELISA and reactive with most HCV antigens by RIBA were also clearly positive, whereas only two eluted samples from the eight HCV RNA-negative patients weakly reactive by ELISA and showing weaker reactivity by RIBA were positive by ELISA. This implied that weakly positive samples from patients with resolved HCV infection could be missed with this procedure, and that prevalence rates based on the DBS method must be considered minimal.


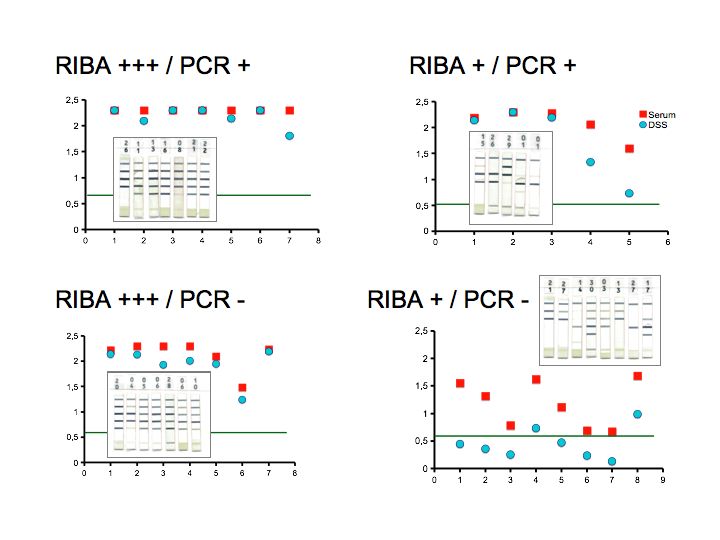


**Legend to figure**

Comparison of absorbance values obtained with various categories of serum samples positive for anti-HCV antibodies, when samples were tested directly as serum (squares) and as DSS eluates (circles). RIBA+++/PCR+: HCV RNA-positive patients strongly reactive with at least three HCV antigens by recombinant immunoblot assay. RIBA+/PCR+: HCV RNA-positive patients strongly reactive with only 1 or 2 HCV antigens by RIBA. RIBA+++/PCR-: HCV RNA-negative patients strongly reactive by ELISA and reactive with most HCV antigens by RIBA. RIBA+/PCR-: HCV RNA-negative patients weakly reactive by ELISA and even more weakly reactive by RIBA. Samples numbers are shown on the x axis, and absorbance values (492 nm) on the y axis. The horizontal bar represents the cut-off value. Inserts: serological profiles of the different samples (same order as the x axis).
